# An altered microbiome in a Parkinson’s disease model *Drosophila melanogaster* has a negative effect on development

**DOI:** 10.1038/s41598-021-02624-1

**Published:** 2021-12-08

**Authors:** Jade Parker-Character, David R. Hager, Tanner B. Call, Zachary S. Pickup, Scott A. Turnbull, Evan M. Marshman, Shaleen B. Korch, John M. Chaston, Gerald B. Call

**Affiliations:** 1 Biomedical Sciences Program, College of Graduate Studies, Midwestern University, Glendale, AZ USA; 2 Arizona College of Osteopathic Medicine, Midwestern University, Glendale, AZ USA; 3 Department of Plant and Wildlife Sciences, College of Life Sciences, Brigham Young University, Provo, UT USA; 4 Department of Pharmacology, College of Graduate Studies, Midwestern University, Glendale, AZ USA

**Keywords:** Microbiome, Neurodegeneration, Drosophila

## Abstract

Parkinson’s disease (PD) is the second most common neurodegenerative disease, besides Alzheimer’s Disease, characterized by multiple symptoms, including the well-known motor dysfunctions. It is well-established that there are differences in the fecal microbiota composition between Parkinson’s disease (PD) patients and control populations, but the mechanisms underlying these differences are not yet fully understood. To begin to close the gap between description and mechanism we studied the relationship between the microbiota and PD in a model organism, *Drosophila melanogaster*. First, fecal transfers were performed with a *D. melanogaster* model of PD that had a mutation in the *parkin* (*park*^*25*^) gene. Results indicate that the PD model feces had a negative effect on both pupation and eclosion in both control and *park*^*25*^ flies, with a greater effect in PD model flies. Analysis of the microbiota composition revealed differences between the control and *park*^*25*^ flies, consistent with many human studies. Conversely, gnotobiotic treatment of axenic embryos with feces-derived bacterial cultures did not affect eclosure. We speculate this result might be due to similarities in bacterial prevalence between mutant and control feces. Further, we confirmed a bacteria-potentiated impact on mutant and control fly phenotypes by measuring eclosure rate in *park*^*25*^ flies that were mono-associated with members of the fly microbiota. Both the fecal transfer and the mono-association results indicate a host genotype-microbiota interaction. Overall, this study concludes functional effects of the fly microbiota on PD model flies, providing support to the developing body of knowledge regarding the influence of the microbiota on PD.

## Introduction

Neurodegenerative diseases, like Alzheimer’s and Parkinson’s disease, create a high burden of morbidity and mortality, which is expected to increase in the next few decades^1,2^. Parkinson’s disease (PD) is the second most common neurodegenerative disease affecting more than ten million people worldwide^2^. Most cases of PD are idiopathic, while a small percentage (3–5%) is genetic in origin^3^. Of these genetic cases, mutation of the *PRKN* gene contributes to approximately 50% of all autosomal recessive juvenile parkinsonism^4^. *Drosophila melanogaster* (the fruit fly) has been used extensively as a model to better understand and characterize many neurodegenerative diseases^5,6^. Mutation of the *Drosophila melanogaster* ortholog of the *PRKN* gene, *parkin (park)*, leads to a tenable model of PD that has many similarities to PD patients: selective loss of dopaminergic neurons, decreased motor function, loss of olfaction, reduced lifespan, mitochondrial dysfunction and others^7–10^. The relative ease of use of flies and powerful genetic tools available in this fly PD model has contributed greatly to the study and understanding of PD^5^.

Gastrointestinal (GI) dysfunctions are among the most common non-motor symptoms associated with PD, with constipation being the most common premotor symptom, affecting more than 70% of PD patients^11^. There have been a number studies that have demonstrated that the gut microbiota is altered in PD patients compared to healthy control individuals^12^ and it has been hypothesized that this altered microbiota is largely responsible for many of the GI disorders observed. Beyond this, the altered PD microbiota has been hypothesized to play a role in non-GI PD symptoms, specifically related to the gut-brain axis. In support of this, PD patient fecal transplant into germ-free PD model mice produced an increase in motor deficits compared to PD model mice with a healthy donor fecal transplant^13^. Taken together, the results from these previous studies prompted our investigation of the microbiota in the *park* mutant fly model.

Relative to humans and other mammals, the *D. melanogaster* microbiota is low-abundance and low-diversity, making it simpler and easier to study microbiota interactions. Laboratory and wild flies are typically colonized by 10^4^–10^5^ microorganisms, and the 2–5 most abundant isolates often represent > 90% of the microbial community^14–16^. The most represented bacteria in the gut are usually acetic acid (AAB) and lactic acid bacteria (LAB), especially members of the genera *Acetobacter* and *Lactobacillus*, respectively, and *Enterobacteriaceae.* Similar to mammals, the fly microbiota composition is determined by both fly genotype and diet^16–19^. Fly larvae possess no gut microbes upon hatching and thus obtain and develop their microbiota from both the environment and food source. Because there is no evidence of high-fidelity host-mediated acquisition or retention of specific microorganism within or across generations, the *Drosophila* microbiota is ‘inconstant’; although some bacterial isolates colonize and persist within the gut better than others^20–22^. A previous analysis of the microbiota in a PD fly model revealed differences in the diversity, but not specific taxonomic changes, between the microbiota of control and PD-model flies^23^. The intracellular endosymbiont *Wolbachia* is also a common inhabitant of the reproductive tract of *Drosophila* and, unlike the gut microbiota, is transmitted from mother to offspring within the egg^24^.

The association between *Drosophila* and its microbiota is experimentally tractable: bacteria-free embryos are readily derived by bleach treatment and members of the *Drosophila* microbiota can be isolated in pure culture in the laboratory. Inoculating bacteria-free fly embryos with a defined microbial species or community is called gnotobiotic culture and permits exquisite dissection of the contributions of individual microorganisms to specific fly phenotypes^25^. Adding back one or more bacterial species to the same genotype of sterile fly embryos permits the detection of the magnitude of variation in host traits that is due to the microbiota^26^. Unlike the gut microbiota, bleach treatment does not eliminate *Wolbachia* from the fly embryos.

In this study we sought to better define the relationship between the microbiota and a *D. melanogaster* PD model by addressing two major questions: (1) Does microbiota manipulation, including via fecal transfer, bacterial-elimination, or gnotobiotic culture affect development, an early and fundamental biologic process, in a PD-fly model? (2) Does the microbiota vary between control and PD model flies? This study aims to address these questions by measuring fly pupation, eclosion, and/or microbiota composition under a variety of conventional and gnotobiotic culture conditions.

## Results

### Fecal transfer from *park*^25^ flies reduces total pupation rates

**Figure 1:**
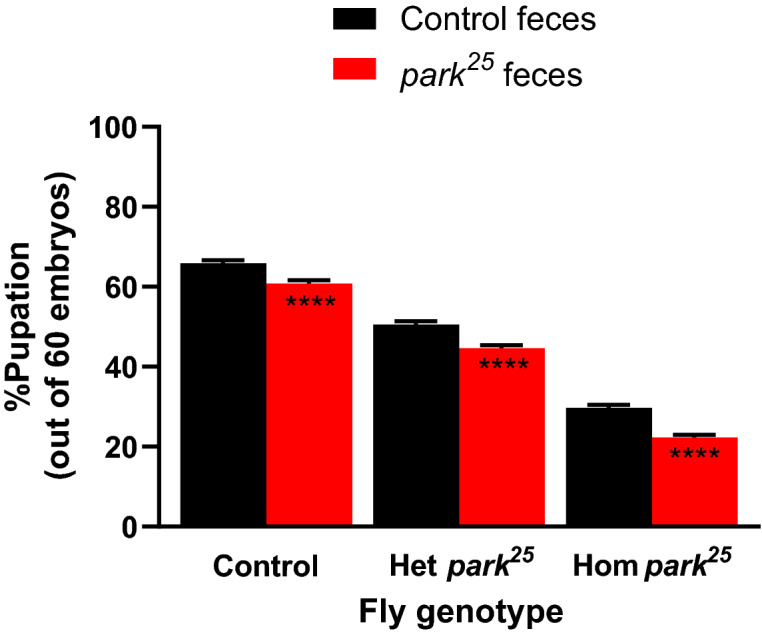
Feces from *park*^*25*^ flies reduces total pupation rate. Total pupation rates were calculated in control, heterozygous (Het) *park*^*25*^ and homozygous (Hom) *park*^*25*^ flies from the 60 embryos that were placed on food that had control or *park*^*25*^ feces present. Data are presented as mean and SEM. Asterisks represent the results of a post-hoc Sidak’s multiple comparisons test comparing the two feces groups within each fly genotype (**** = *P* < 0.0001). Results are from 45 separate vials in each group.

To determine whether differences in the microbiome between control and *park*^*25*^ flies might contribute to variation in fly phenotypes we compared the pupation rate of flies that received fecal transfers from control and *park*^*25*^ donors. Fecal transfers were performed by allowing males to defecate on cooked but not autoclaved diet vials for 3 days before transferring fly embryos to the feces-seeded diet. The *park*^*25*^ feces reduced the total pupation rate of all fly genotypes when compared to the embryos that were placed on food that contained control feces (post-hoc Tukey test: *P* < 0.0001 for all three; Fig. 1). Each of the pupation rates are based off the 60 embryos placed on the food, such that both the *park*^*25*^ heterozygous and homozygous pupae numbers come from the same 60 embryos. Two-way ANOVA analysis revealed that 86% of the variation is due to genotype (*P* < 0.0001), which is expected given the effects of the *park*^*25*^ mutation. Additionally, 3.6% of the variation is due to the fecal transfer effect (*P* < 0.0001). When analyzing daily pupation rates, the homozygous *park*^*25*^ flies were the only genotype that had reduced pupation rates on two consecutive days when they received feces from *park*^*25*^ mutants, suggesting that the *park*^*25*^ homozygous flies are more susceptible to the detrimental effects of the *park*^*25*^ fecal transfer (Supplementary Fig. S1). Together, these results identify a negative effect on flies of multiple genotypes when they received a fecal transfer from *park*^*25*^ versus control flies.

### The *park*^25^ feces reduces fly eclosion rates

**Figure 2:**
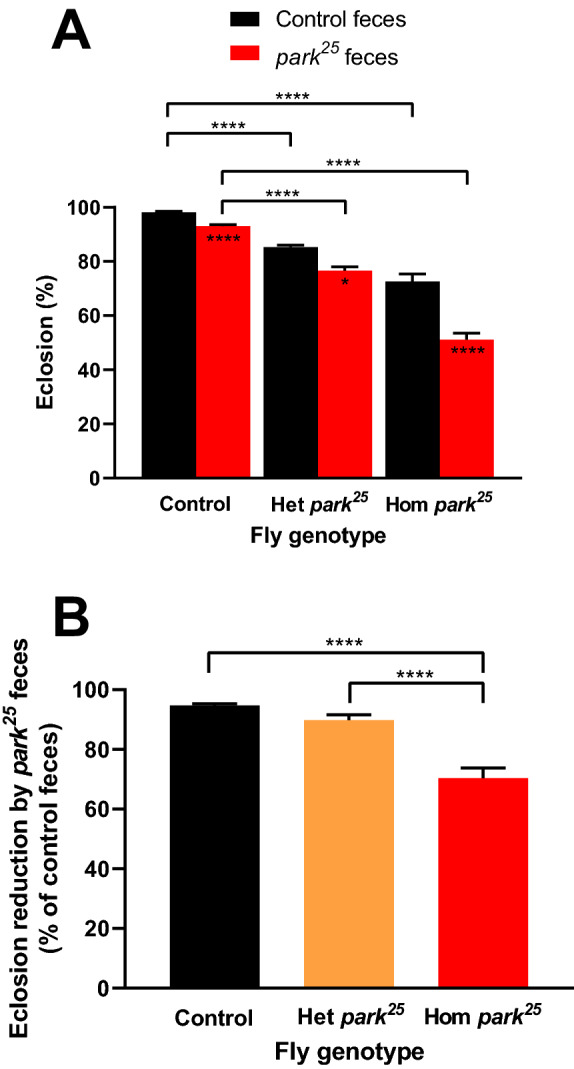
Eclosion rates are reduced in all fly genotypes but more in homozygous *park*^*25*^ flies with *park*^*25*^ feces transfer. (**A**) Total eclosion rates were determined in control, heterozygous (Het) *park*^*25*^ and homozygous (Hom) *park*^*25*^ flies based on the total number of pupae for each genotype. (**B**) The relative eclosion reduction caused by the *park*^*25*^ fecal transfer, calculated as a percentage of the control feces eclosion, was determined. Data are presented as mean and SEM. Asterisks represent the results of a post-hoc Tukey’s multiple comparisons test. Asterisks inside the bars compare the two feces groups within each fly genotype. * = *P* < 0.05 and **** = *P* < 0.0001. Results are from 45 separate vials in each group.

Eclosion rates for each fly genotype-fecal transfer combination were determined by dividing the number of flies that eclosed by the total number of pupae that had developed for that genotype. Figure 2A shows that all three fly genotypes experienced a reduced eclosion rate when placed on the *park*^*25*^ feces compared to the control feces (control and homozygous *park*^*25*^, *P* < 0.0001; heterozygous *park*^*25*^, *P* = 0.0101). As with pupation, the majority of variation identified was due to fly genotype (54%, *P* < 0.0001), which is reflected by the reduced eclosion rates of both the *park*^*25*^ heterozygous and homozygous flies on control feces compared to the control flies on control feces (both *P* < 0.0001). In agreement with pupation, a smaller amount of variation was due to the feces (11.9%, *P* < 0.0001) and there was also a significant interaction between the fly genotype and the feces transfer, indicating that there might be a specific effect of the fecal transfer in the *park*^*25*^ fly (*P* = 0.0042, 1.4% of variation).

Regardless of the source of the fecal inoculum, both the heterozygous and homozygous *park*^*25*^ flies had reduced eclosion rates compared to the control flies, indicating that the *park*^*25*^ genotype likely has a reduced eclosion rate due to the *park*^*25*^ mutation (Fig. 2A). However, it appears that the *park*^*25*^ fecal transfer had an additional negative impact on the *park*^*25*^ flies. This feces-dependent differential effect, based on fly genotype, of eclosion reduction due to the *park*^*25*^ feces becomes more apparent when observing the number of pupae that failed to eclose. This measurement indicates that the control flies did not have an increased number of failed eclosures due to *park*^*25*^ feces (*P* = 0.1423), while the heterozygous and homozygous *park*^*25*^ pupae did (*P* = 0.0023, *P* < 0.0001, respectively; Supplementary Fig. S2).

Further, the detrimental effects of the *park*^*25*^ feces on fly eclosion were of larger magnitude for the homozygous *park*^*25*^ flies than other genotypes. When we calculated the percent eclosion rates of each genotype on *park*^*25*^ feces relative to control feces, there was no difference in the eclosion rates of the heterozygous mutants and control flies (*P* = 0.1211), but both genotypes had higher eclosion rates than the *park*^*25*^ homozygous flies (*P* < 0.0001 vs both, Fig. 2B). Further support of differential feces-genotype interaction is provided by observing the eclosion rate over time. Supplementary Figure S3 shows that the control flies experienced a reduction in eclosion due to *park*^*25*^ feces on day 10 (*P* < 0.0001), while heterozygous *park*^*25*^ flies had no significant reduction on any day in the experiment. However, homozygous *park*^*25*^ flies experienced a reduction in eclosion due to *park*^*25*^ feces on days 9, 10 and 11 (*P* = 0.0001, *P* = 0.0012, *P* = 0.0005, respectively).

### The whole-body microbiota varies between conventional PD model and control flies

**Figure 3:**
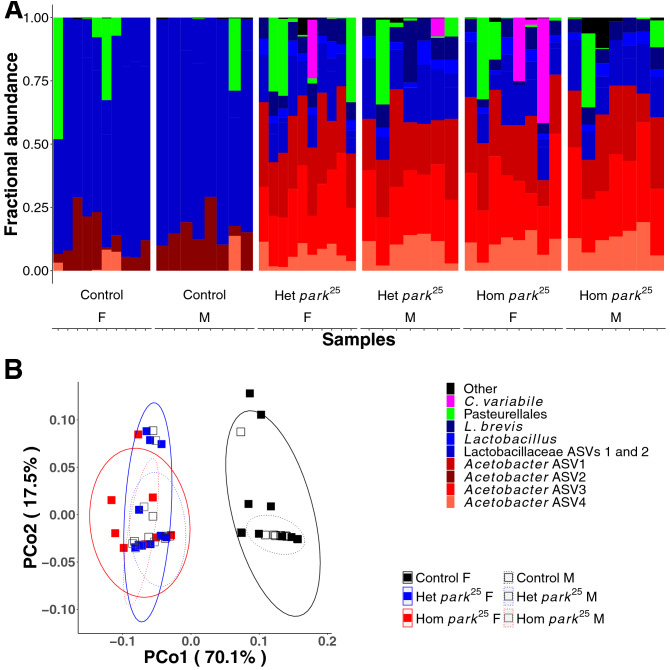
The microbiota of control and *park*^*25*^ flies. (**A**) Taxon plot of control and *park*^*25*^ flies, separated by sex. Mutants of *park*^*25*^ were distinguished as homozygotes and heterozygotes based on the presence of the Tubby marker. Bars represent distinct ASVs. The legend shows the lowest taxonomic level that was assigned to each ASV. (**B**) Principal coordinates plot, showing the first two coordinates calculated from a weighted Unifrac distance matrix.

Our observations suggest that the composition of *park*^*25*^ and control fly microbiomes are different and cause different developmental effects on the flies tested. Thus, as an extension of these results we measured the bacterial microbiota of whole-body conventionally reared *park*^*25*^ and control flies. The samples for sequencing were collected at a different time than the experiments above and, because of the inconstant microbiota^27^, the sequencing results should not be conflated as measuring the microbiota of the flies that deposited feces in the previous experiments. Sequencing of the V4 region of the 16S rRNA gene revealed significant differences in the whole-body microbial communities of our stocks. The most notable difference between the mutant and control populations was the presence of the reproductive tract endosymbiont *Wolbachia* in the control flies (Supplementary Fig. S4 and Supplementary Table S1); however, *Wolbachia* are not likely to be transferred between flies via ingestion and therefore are not likely candidates for the effects observed with fecal transfer. After *Wolbachia* were removed from the analysis (to focus on non-reproductive tract microorganisms), beta-diversity metrics that factor microbial abundance reported significant differences in the microbiota composition of the different fly stocks with fly genotype, but not with the sex of the flies (Fig. 3, Supplementary Fig. S5, and Table 1). Also, there was not a significant genotype-sex interaction, indicating that both males and females showed the same genotype-dependent changes in microbiota composition (Table 1). Amplicon sequence variants assigned to the LAB (more abundant in controls) and AAB (less abundant in controls) were significantly different in relative abundance between flies of different genotypes (Supplementary Fig. S6). The decreased abundance of AAB in the control flies, which also bore *Wolbachia*, is consistent with previous reports that *Wolbachia* prevalence is negatively associated with AAB abundance^28^. Overall, the data reveal a consistent difference in the microbiota composition of *Wolbachia*-discordant control and *park*^*25*^ mutant flies that were reared side-by-side under conventional laboratory conditions.

**Table 1:** Genotype and sex-dependent differences in microbiota composition of control and *park*^*25*^ mutants.

	df	Weighted Unifrac					Unweighted Unifrac					Bray Curtis				
		SS	MS	F	R^2^	P	SS	MS	F	R^2^	P	SS	MS	F	R^2^	P
G	2	0.37	0.19	72.18	0.71	0.001	0.20	0.10	6.12	0.21	0.001	6.71	3.36	111.90	0.79	0.001
S	1	0.00	0.00	1.38	0.01	0.24	0.02	0.02	1.25	0.02	0.29	0.04	0.04	1.33	0.00	0.25
V	3	0.04	0.01	5.02	0.07	0.002	0.04	0.01	0.90	0.05	0.54	0.47	0.16	5.27	0.06	0.001
GS	2	0.00	0.00	0.80	0.01	0.49	0.02	0.01	0.73	0.02	0.70	0.02	0.01	0.41	0.00	0.79
R	41	0.11	0.00	0.20			0.67	0.02	0.70			1.23	0.03	0.15		
T	49	0.53	1.00				0.96	1.00				8.48	1.00			

Effects of fly genotype (G), fly sex (S), fly vial (V), and the G-S interaction (GS) are shown along with residuals (R) and totals (T) as determined by PERMANOVA. PERMANOVA values are degrees of freedom (df), sum of squares (SS), mean squares (MS), F statistic (F), *R*^2^ value (*R*^2^), and *P*-value (*P*).

### Axenic preparation of *park*^25^ homozygous embryos has a dramatic effect on eclosion

**Figure 4:**
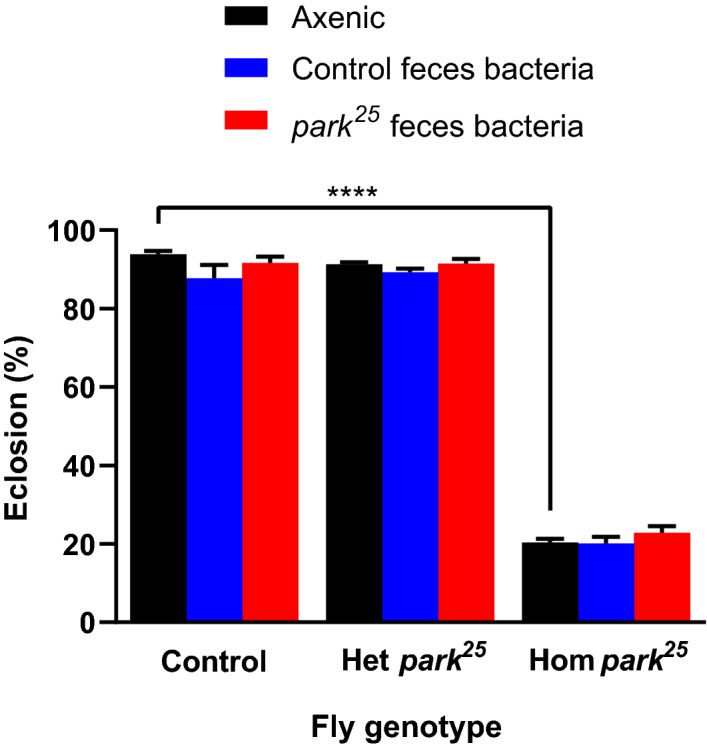
Axenic preparation of homozygous *park*^*25*^ embryos dramatically reduces eclosion rate. Embryos from *park*^*25*^ and control flies were made axenic and gnotobiotic for feces-derived bacteria from *park*^*25*^ or control flies. Pupae count and eclosion was recorded from each vial. Heterozygous (Het) *park*^*25*^ pupae were differentiated from the homozygous (Hom) *park*^*25*^ pupae by the presence of the Tubby marker on the TM6C balancer chromosome. Data are presented as mean and SEM. Post-hoc Tukey’s analysis results are shown: **** = *P* < 0.0001. Sample (number of vials) sizes: Control (axenic = 40, control feces = 41, *park*^*25*^ feces = 42), Het and Hom *park*^*25*^ (axenic = 202, control feces = 63, *park*^*25*^ feces = 59).

To determine if the different effects of fecal transfer from *park*^*25*^ or control flies could be attributed to variation in the bacterial microbiota we measured eclosion rates in flies that were inoculated as sterile embryos with cultured feces from control and *park*^*25*^ adult flies. We observed two major differences between the different treatment approaches: fecal transfer vs fecal bacterial culture inoculation. First, the process of generating axenic embryos dramatically decreased the eclosion rates of homozygous, but not heterozygous, *park*^*25*^ mutants relative to controls. (Fig. 4, *P* < 0.0001). Second, there was no effect related to feces source (control or *park*^*25*^) that were used to create the bacterial cultures on eclosion rate regardless of the genotype that received the bacterial culture. These results were not due to a limited number of homozygous pupae present in the tubes, as all experimental vials with *park*^*25*^ pupae contained approximately 30% *park*^*25*^ homozygous pupae, while the axenic experimental vials had the most pupae/vial (Supplementary Table S2). The most significant source of variation was fly genotype (*P* < 0.0001), accounting for 65% of the variation, with the bacterial status of the fly contributing only 0.23% to variation (*P* = 0.0082). No difference in the eclosion rates were observed between the axenic and the two gnotobiotic treatments regardless of fly genotype (all *P* > 0.52). Together, these results suggest that variation in the cultured bacteria in fly feces does not contribute to the variation in the eclosion rates observed in *park*^*25*^ mutant and control flies when reared on fly feces-seeded vials.

**Figure 5:**
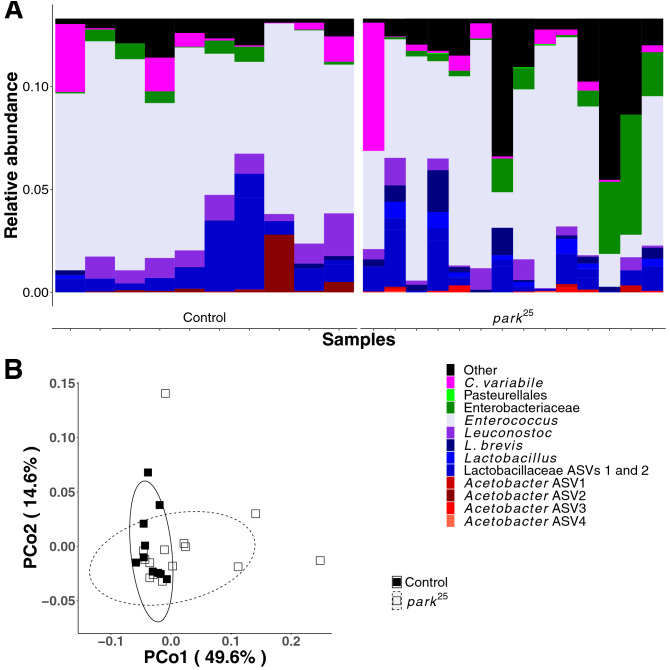
The microbiota of feces from control and *park*^*25*^ flies. Fecal samples were collected from male controls and a mixture of heterozygous and homozygous male park^25^ flies. (**A**) A taxon plot with bars representing distinct ASVs. The legend shows the lowest taxonomic level that was assigned to each ASV. (**B**) Principal coordinates plot, showing the first two coordinates calculated from a weighted Unifrac distance matrix.

In contrast to whole-body flies, the major difference in the microbiota of fecal samples collected from *park*^*25*^ mutant and control flies was attributed to microbial identity, not abundance (Table 2 where unweighted Unifrac, but not weighted Unifrac or Bray–Curtis distance metrics showed significant variation in the community composition with host genotype; corresponding PCoA plots are in Fig. 5 and Supplementary Figure S7. Analyzed feces were collected from independent experiments and, because of the inconstant microbiota, the results cannot be directly compared to other experiments here. Analysis of the fecal samples and siblings of the fecal donors revealed that the microbiota composition varied with respect to both the host genotype and the sample type (fly or feces). For example, the feces was dominated by an *Enterococcus* ASV (Fig. 5A) and the four AAB ASVs that were most abundant in the flies (Fig. 3, Supplementary Table S3 and Supplementary Fig. S8) were detected at very low levels. The low level of AAB reads in the feces suggests that AAB DNA in living or dead cells persists poorly between the location of abundant bacterial cells in the flies and collection of < 24 h old feces. Additionally or alternatively, *Enterococcus* cells may grow rapidly in the feces since there is little evidence of their abundance in live flies, or *Enterococcus* DNA may survive gut transit well. Together, these results suggest that the role of *P* generation defecation in establishing the F1 adult microbiota in the flies in our study, and perhaps flies generally, is incompletely understood.

**Table 2:** Genotype-dependent differences in fecal microbiota composition of control and *park*^*25*^ mutants.

	df	Weighted Unifrac					Unweighted Unifrac					Bray-Curtis				
		SS	MS	F	R^2^	P	SS	MS	F	R^2^	P	SS	MS	F	R^2^	P
G	1	0.02	0.02	2.34	0.09	0.061	0.38	0.38	2.60	0.10	0.003	0.24	0.24	1.82	0.08	0.10
V	10	0.11	0.01	1.24	0.46	0.26	1.54	0.15	1.05	0.42	0.36	1.16	0.12	0.89	0.39	0.61
R	12	0.10	0.01	0.45			1.76	0.15	0.48			1.57	0.13	0.53		
T	23	0.23	1.00				3.69	1.00				2.96	1.00			

Effects of fly genotype (G), fly vial (V), residuals (R), and totals (T) as determined by PERMANOVA. PERMANOVA values are degrees of freedom (df), sum of squares (SS), mean squares (MS), F statistic (F), *R*^2^ value (*R*^2^), and *P*-value (*P*).

### Variation in the bacterial microbiota of *D. melanogaster* influences eclosion success in *park*^25^ mutants

**Figure 6:**
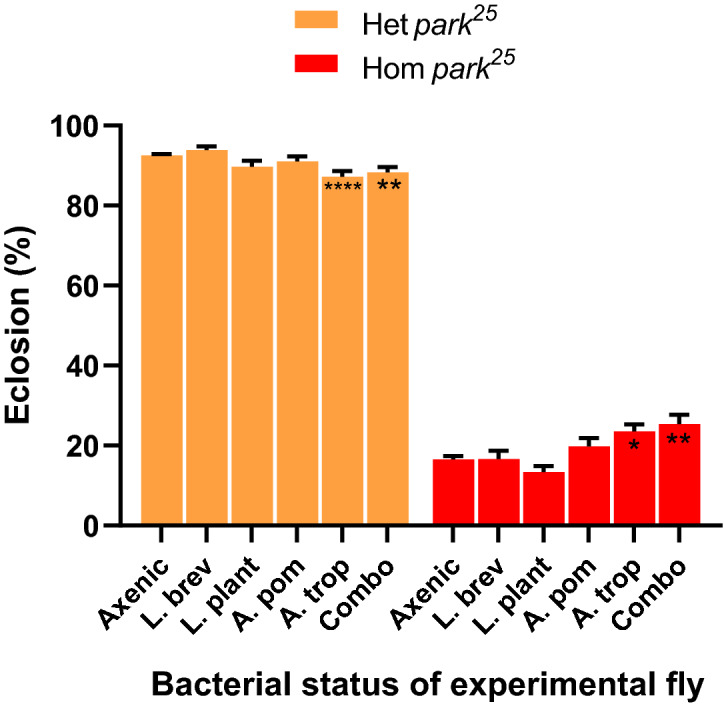
Mono-association with *A. tropicalis* or a combination of bacteria with *park*^*25*^ flies can alter eclosion rate. Embryos from *park*^*25*^ flies were made axenic and then mono-associated with four different laboratory bacterial strains, or inoculated with an equal CFU combination of the four strains (Combo). Pupae count and eclosion was recorded from each vial. Heterozygous (Het) *park*^*25*^ pupae were differentiated from the homozygous (Hom) *park*^*25*^ pupae by the presence of the Tubby marker on the TM6C balancer chromosome. Data are presented as mean and SEM. Post-hoc Dunnett’s analysis comparing to the axenic flies are shown: **** = *P* < 0.0001, ** = *P* < 0.01, * = *P* < 0.05. Axenic (*n* = 160), L. brev = *Lactobacillus brevis* (*n* = 24), L. plant = *Lactiplantibacillus plantarum* (*n* = 37), A. pom = *Acetobacter pomorum* (*n* = 35), and A. trop = *Acetobacter tropicalis* (*n* = 37), combination (*n* = 32).

To understand the extent to which bacterial microbiota of *D. melanogaster* influence eclosion success in homozygous and heterozygous *park*^*25*^ mutants, we compared eclosion rates of mono-associated flies. Previous gnotobiotic experiments provided no direct evidence that variation in the bacterial communities of the flies influenced fly eclosion in these *park*^*25*^ mutants. In contrast, fecal transfer experiments suggested that microbiome changes did affect fly eclosion. Similar to our results with cultured feces, we observed dramatically reduced eclosion in homozygous *park*^*25*^ flies compared to heterozygous *park*^*25*^ flies (Fig. 6). Unlike with cultured feces, there were differences in fly eclosion rates when they were axenic or colonized with a combination of four bacterial species cultured from flies in Ithaca, NY^29^ (heterozygous *park*^*25*^: *P* = 0.0025, homozygous *park*^*25*^: *P* < 0.0001) or individually with *Acetobacter tropicalis* (heterozygous *park*^*25*^: *P* = 0.0088, homozygous *park*^*25*^: *P* = 0.0456). Additionally, there was a significant fly genotype-bacterial treatment interaction (two-way ANOVA, *P* < 0.0001): the combination and *A. tropicalis* treatments led to higher eclosion survival than the axenic treatment in *park*^*25*^ homozygous flies; but lower survival than axenic flies for *park*^*25*^ heterozygous flies. Taken together, these results confirm that variation in the bacterial microbiota of *park*^*25*^ flies can contribute to variation in a key survival phenotype, eclosion success.

## Discussion

There have been many studies showing alterations in the fecal microbiota of PD patients compared to control populations (reviewed in^12^). To our knowledge, there has only been a single study that has looked at the microbiota in a fly PD model (*PINK1*^*B9*^) that also identified differences between the microbiome of the PD model and control flies^23^. Under condition-matched conventional rearing, our *park*^*25*^ PD model fly microbiota was considerably different from the control fly (Fig. 3), with significant differences in the abundance of AAB and LAB. Fly sex was not a determinant of variation in microbiota composition. Alterations in microbiota were observed in conventionally-reared flies and therefore factors such as inconstant exposure to or acquisition of distinct sets of microbes in the different vials from which samples were drawn could contribute to the observed effects; though the level of replication and matched rearing conditions of the flies suggests potential influences of host genotype on fly microbiota composition. Future experiments with gnotobiotic flies could conclusively rule out environmental effects but could be challenging because of the low survival rates of the homozygous *park*^*25*^ flies (Fig. 4).

Presence of the endosymbiont *Wolbachia* has been associated with lower counts of *Acetobacter spp.* in other flies^30^ and is positively correlated with worsening phenotypes in a fly model of Alzheimer’s disease. In agreement with this, a recent report linked *Wolbachia* and neurodegenerative disease severity in *Drosophila* by showing that administration of a *Lactobacillus* probiotic increased *Acetobacter* abundance, lowered *Wolbachia* titers, and ameliorated Alzheimer’s disease phenotypes^31^. Thus, the presence of *Wolbachia* could be a factor contributing to differences between the microbiota of our control and *park*^*25*^ flies. Our analysis identified that the *Wolbachia* status of our control and mutant stocks was not congruent. In consideration of the fecal transfer, it is important to recognize that *Wolbachia* are intracellular endosymbionts that are transmitted between generations via the germ line and not fecal transfer^24^. This was validated by sequence analysis which demonstrated that *Wolbachia* were not represented in the sequenced fecal samples, confirming their absence and irrelevance to observed functional effects of the *park*^*25*^ fecal transfer (Fig. 5). Our data indicate that *Acetobacter spp.* are reduced in our control flies both in diversity and abundance (Fig. 3 and Supplementary Fig. S6). The data do raise the question if the differences in the eclosion of control and *park*^*25*^ flies is due to their discordant *Wolbachia* status. While some influence on eclosion is possible, *Wolbachia* is unlikely to be the sole contributor to this observed difference as PD phenotypes have been detected in another laboratory using the same *park*^*25*^ mutant and a *Wolbachia-*concordant control strain (^8^, unpublished data). The potential direct and indirect (through the microbiota) influence(s) *Wolbachia* has on *park*^*25*^ mutant development requires further analysis.

We used fecal transfers to assess whether the microbiota influences *park*^*25*^ mutant eclosion success. Microbiota studies typically rear dechorionated embryos free of bacteria or with a defined bacterial inoculum^25^, but we adopted an alternate fecal transfer approach for two reasons. First, fecal transfers have successfully identified microbiota effects in other studies (e.g., ^32^) and provided a straightforward method to use in initial functional explorations. Second, as shown in Figs. 4 and 6, the viability of axenic and gnotobiotic *park*^*25*^ flies is very low, which makes this process extremely difficult and impedes experimentation. We do not know the cause of this high mortality rate, but it appears to be related to the dechorionation process. Alternative approaches that avoid the dechorionation step are available, but these approaches also have limitations. For example, while raising axenic fly stocks for several generations after dechorionation the stocks are vulnerable to bacterial contamination, requiring the use of antibiotics which can alter but not necessarily eliminate all colonizing microorganisms. One successful recent approach fed bacteria to newly eclosed (and presumably bacteria-depleted) *PINK1* mutant flies^23^, which might be a more high-throughput approach. Further understanding of the mechanism that determines the difference in microbiota composition between control and *park*^*25*^ flies may provide insight into why the microbiota composition of adult flies and fecal transfers are distinct.

The exposure of hatching larvae to *park*^*25*^ fly feces led to dramatically reduced fly eclosion success than did exposure to control feces. Conversely, when we inoculated flies with cultured feces there was no difference in the effect on fly eclosion. The difference in outcome between the two experiments suggests that different effectors are transmitted, or possibly diluted, when the feces is cultured first versus when it is directly deposited. We hypothesized that the fecal microbiota would largely reflect the adult fly microbiota and that culturing feces versus direct deposition would lead to similar outcomes. However, we detected no difference between the fecal microbiota of mutant and control flies even though adult mutant and control flies had a different microbiota composition. In this regard, the outcomes of the fecal transfer vs culture experiments were congruent: there was only a difference in recipient phenotypes when there was a difference in the source’s microbiota composition. Altered phenotypes following the transfer of direct but not cultured feces could be also be due to an effector that is abiotic or non-bacterial (e.g., fungal); or it may be that the culture step abates the effect. Culture in standard laboratory medium may select for certain strains in ways that does not occur in the fly diet, leading to differences in identity and abundance of key microbiota members. To address these potential limitations, the bacterial mono-association experimental approach was critical to understanding whether variation in bacterial microbiota can alter the eclosion success of *park*^*25*^ flies in a genotype-dependent (heterozygous vs. homozygous) manner.

We found that there is a functional consequence with the feces transfer, in that the *park*^*25*^ feces had a negative impact on pupation and eclosure on both the control and *park*^*25*^ flies; however, the homozygous *park*^*25*^ flies appeared to be affected the most. It is possible that homozygous *park*^*25*^ flies are more susceptible to *park*^*25*^ feces due to their general weak state. It is established that *park*^*25*^ flies have reduced mitochondrial function and deficiency in energy production^8,10^. Additionally, axenic flies have disrupted insulin-like signaling and glucose regulation compared to microbiota-colonized flies^26,33,34^. Therefore, axenic homozygous *park*^*25*^ flies may have compounding, additive deficiencies that reduce their ATP production during eclosion, which is likely a high energy-requiring process. In support of this idea, 34.9% of all axenic *park*^*25*^ pupae were found dead and stuck in the process of eclosing, compared to 1.6% of the axenic control flies or 3.4% of the axenic heterozygous *park*^*25*^ flies. Moreover, when the *park*^*25*^ homozygous flies had two fecal-derived gnotobiotic treatments, the rate of being stuck in eclosure reduced to 15.9% with the control bacteria and 13.4% with the *park*^*25*^ bacteria. This reduced rate of incomplete eclosion in the gnotobiotic populations might have masked the negative effect of the *park*^*25*^ bacteria on eclosure in these flies compared to the axenic controls, however, since both gnotobiotic groups had similar reductions in getting stuck, this is unlikely.

Despite the large number of studies showing an altered fecal microbiome in PD patients, there have been very few studies demonstrating that the PD microbiome has functional consequences. The most compelling functional study utilized PD model mice that had a fecal transplant from PD and healthy control patients. The PD microbiome transplant mice showed an increase in motor dysfunction and alpha-synuclein aggregation^13^. Our fecal transfer experiments, like another recent *Drosophila* PD model functional microbiome study^23^, did not directly manipulate the microbiome to a specifically-defined microbiota composition but we do use mono-association experiments to demonstrate species-specific influences of the associated microorganisms. Our study is the first to indicate that there might be a specific microbiota-fly genotype effect that might also be occurring with the PD microbiome and homozygous *park*^*25*^ flies. This type of specific microbiota-fly genotype interaction is known to happen with *Wolbachia*^35^. The current study adds to the small group of publications that indicate that the altered PD microbiome negatively affects biological processes in the host. This provides further support for research and identification of bacterial species involved in these functional effects, which has the potential to direct microbiome manipulation in PD patients that may alleviate symptoms.

## Methods

### *Drosophila* stocks and maintenance

Mutant, *park*^*25*^, *Drosophila melanogaster* were provided by Dr. Leo Pallanck at the University of Washington. This mutant stock was derived from *w*^*1118*^ control flies, which were obtained from the Bloomington Drosophila Stock Center (Indiana University). In all experiments, *w*^*1118*^ flies were used as the control for the *park*^*25*^ flies. The *park*^*25*^ stock in our laboratory has been backcrossed with the *w*^*1118*^ stock so that all chromosomes are from the *w*^*1118*^ background. The *park*^*25*^ chromosome is balanced over the TM6C balancer, allowing for identification of homozygous and heterozygous flies through use of the *Tubby* gene phenotype. All fly stocks were raised on standard cornmeal-molasses diet at 25 °C in a 14/10-h light cycle.

### Fecal transfer

In each of three separate experiments, five separate food vials for each fly genotype were seeded with forty males of that genotype to allow flies to deposit their feces on the food. Males were used so that no embryos were laid on the food. All males were over the age of three days to ensure that they had an established microbiome^36^. A random mix of both homozygous and heterozygous *park*^*25*^ male flies were used as the fecal donors for the *park*^*25*^ feces. Four days post-seeding, 60 embryos of the specific genotype were placed on the feces-prepared food. The embryos were collected and counted as described below.

### Embryo collection

Fly stocks (control and *park*^*25*^—heterozygous and homozygous) were placed in square polypropylene fly bottles with a molasses “puck” as the lid. The molasses puck was a 35 mm petri dish cover filled with a molasses-agar media (200 mL ddH_2_O, 6.24 g drosoagar [Genesee Scientific], 2 mL Tegosept, and 50 mL of molasses). The bottles were stored upside down, so the puck was at the bottom, while the top of the bottle had small holes for air transfer. Yeast paste was put on the inside of the bottle to help stimulate oogenesis. Flies were allowed to lay their embryos for < 24 h at 25 °C. Embryo collection was performed by wetting a paintbrush with ddH_2_O and carefully brushing the embryos from the molasses-agar. The embryos were then washed off the puck directly into a 1.5 mL centrifuge tube and were rinsed with ddH_2_O for a total of three washes. After washing, the embryos were pipetted into a glass spot plate in one of the three wells. Under a stereo microscope, 60 embryos were counted and placed in one of the empty wells with < 0.5 ml water. A fine-tipped paintbrush was used to paint the 60 embryos onto the surface of the fecal-prepared food. A different paintbrush was used for each fly genotype to prevent bacterial transfer between genotypes during embryo deposition. The embryos were collected for six days, with new parental fly populations being introduced every two days to produce three biological replicates with each biological replicate having two technical replicates.

### Axenic and gnotobiotic experiments

We reared flies with bacteria cultured directly from fly feces beginning with axenic fly embryos. Axenic embryos were derived as described previously^25^. Briefly, control and *park*^*25*^ embryos were collected as above and suspended in a 0.6% sodium hypochlorite solution for 2.5 min. These embryos were then transferred to fresh 0.6% sodium hypochlorite solution in a sterile hood to dechorionate the embryos. The sterile, dechorionated embryos were collected with a sterile paintbrush and approximately 60 embryos were brushed onto sterile food. These embryos were either maintained as axenic or inoculated with 5 × 10^5^ CFUs from control or *park*^*25*^ fecal bacterial cultures, or from individual bacterial strains, including, *Lactobacillus brevis*, *Lactiplantibacillus plantarum*, *Acetobacter pomorum*, or *Acetobacter tropicalis*.

To produce the fecal bacterial cultures, feces were collected from the *park*^*25*^ and control fly embryo collection bottles by scraping the feces off with a sterile toothpick to inoculate Luria–Bertani (LB) and modified deMan-Rogosa-Sharpe (mMRS) medium. These cultures were grown at 30 °C with aeration for 16 h. To preferentially cultivate aerotolerant microbes, separate MRS cultures were grown in loosely capped tubes with no shaking for 16 h. To generate the microbiome inoculum, each culture was normalized to 10^7^ mL^-1^, combined in equivalent ratios, and 50 μl containing 5 × 10^5^ total CFUs was used to inoculate the sterile embryos. Control axenic embryos were collected each day for four days with a minimum of eight tubes/day, while the axenic *park*^*25*^ embryos were collected each day for five days with a minimum of 28 tubes/day. The bacterial culture embryos were collected each day for four days with a minimum of 10 tubes/day for control embryos or 14 tubes/day for the *park*^*25*^ embryos.

To confirm axenic flies were truly bacteria-free, pools of five whole-body adult axenic flies from each axenic control vial were homogenized at the end of each experiment and cultured on LB and duplicate MRS plates (one incubated with standard atmospheric conditions, one in microoxic conditions in a sealed, CO_2_-flooded chamber) at 30 °C. If > 10 CFUs/fly were detected, those flies were deemed non-axenic and removed from the analysis.

### Pupation and eclosure measurements

Newly developed pupae were counted on days 5, 6, 7 and 8 post-embryo collection. Due to the *Tubby* mutation on the TM6C balancer chromosome present in the heterozygous *park*^*25*^ flies, homozygous and heterozygous *park*^*25*^ flies were differentiated. Although analyzed separately, these two pupal populations account for the full 60 *park*^*25*^ embryos painted in the fecal transfer experiments. Individual fly eclosion was quantified on days 9, 10, 11 and 12 post-embryo collection.

### 16S sequencing and analysis

We prepared and analyzed DNA samples for 16S rRNA marker gene analysis as done previously^28,30^. Sequencing libraries were prepared by extracting DNA from pools of 10 flies using the Zymo Quick-DNA fecal/soil microbe kit (D6011, Zymo, Irvine, CA). Then, the V4 region of the extracted DNA was amplified and sequenced using a dual-barcoding method described by Kozich^37^, with the exception of substituting Accuprime PFX DNA polymerase reagents for Accuprime PFX Supermix. The Invitrogen SequalPrep Normalization kit was used to normalize samples into pools of 96 samples (in some cases, the samples were normalized as part of a pool with samples not published in this study). Then, fragments in the size range of 250–450 nucleotides were size-selected using a BluePippin (BYU DNA Sequencing Center). Finally, samples in this study were sequenced on partial lanes of a MiSeq using 500 cycle chemistry (paired-end 2 × 250, BioDesign Institute at Arizona State University).

Sequenced reads were analyzed using QIIME2^38^ and R. The reads were trimmed based on quality scores, denoised and dereplicated using DADA2^39^ to call individual amplicon sequence variants (ASVs), and taxonomy was assigned using the GreenGenes classifier^40,41^. To enable the calculation of Unifrac beta-diversity metrics^42,43^, a phylogeny of all ASVs was constructed^44^ based on mafft alignment^45^. For some analyses, *Wolbachia* reads were pre-filtered out so that reproductive tract symbionts were not included in the analysis. Before performing beta-diversity analyses, samples were normalized to varying read thresholds that maximized the number of reads per sample and the number of samples retained: 350 (Fig. 3), 3000 (Fig. 5), and 399 (Supplementary Fig. S8). Permutational multivariate analysis of variance (PERMANOVA) of Bray Curtis distances and of unweighted and weighted Unifrac distances were used to test for host genotype and sex-dependent variation in microbiota composition^46^. We also used Analysis of Composition of Microbiomes (ANCOM) to test for differences in the abundances of specific individual or groups of ASVs^47^.

One sample was removed from Fig. 3 analyses because it was almost exclusively enterococcus. Removing it did not change the significance of any comparisons but did reduce noise. Analyses of the data that include this sample are presented in Supplementary Figure S4.

### Development statistical analysis

Statistical analyses were performed by using One-Way and Two-Way ANOVA, with post-hoc Tukey’s, Sidak’s, or Dunnett’s tests to determine differences between the arcsin transformed percentages of each group by GraphPad Prism 9. All other data analysis was in RStudio version 1.3.1093 using R version 3.6.3 or the terminal. All graphs display the mean ± the standard error of the mean. Details on each test performed and their results are presented in the results section or legends.

## Supplementary Information

Supplementary Information.

## Data Availability

The reads are publicly available at the NCBI SRA under Accession number PRJNA776269.

## Code availability

The code to analyze the reads can be accessed as an R tutorial by running ‘devtools::install_github ("johnchaston/JPCparkTutorial")’ and ‘learnr::run_tutorial ("mainTutorial", "JPCparkTutorial")’ in an R console where devtools and learnR, plus other packages executed in the tutorial, are installed.
