# Supplementary material for: An altered microbiome in a Parkinson’s disease model Drosophila melanogaster has a negative effect on development
Source: Sci Rep. 2021 Dec 8;11:23635. doi: 10.1038/s41598-021-02624-1 (PMC8654912; doi:10.1038/s41598-021-02624-1)
Supplement: Supplementary file 1 — Supplementary Information. [file 41598_2021_2624_MOESM1_ESM.pdf]

Supplementary Information

An altered microbiome in a Parkinson's disease model *Drosophila melanogaster* has a negative effect on development

Jade Parker-Character[1], David R. Hager[2], Tanner B. Call[2], Zachary S. Pickup[1], Scott A. Turnbull[2], Evan M. Marshman[3], Shaleen B. Korch[4], John M. Chaston[3], Gerald B. Call[4]*

[1]Biomedical Sciences Program, College of Graduate Studies, Midwestern University, Glendale, AZ, USA

[2]Arizona College of Osteopathic Medicine, Midwestern University, Glendale, AZ, USA

[3]Department of Plant and Wildlife Sciences, College of Life Sciences, Brigham Young University, Provo, UT, USA

[4]Department of Pharmacology, College of Graduate Studies, Midwestern University, Glendale, AZ, USA

*Corresponding author, gcall@midwestern.edu

Supplementary Table S1. Statistics of whole-body microbiota composition differences of control and *park*^{25} mutants.

Effects of fly genotype (G), fly sex (S), and the G * S interaction (GS) are shown along with residuals (R) and totals (T) as determined by PERMANOVA. PERMANOVA values are degrees of freedom (df), sum of squares (SS), mean squares (MS), F statistic (F), R^2 value (R^2), and P-value (P).

		Bray Curtis					Unweighted Unifrac					Weighted Unifrac				
	df	SS	MS	F	R^2	P	SS	MS	F	R^2	P	SS	MS	F	R^2	P
G	2	9.97	4.98	180	0.88	0	0.42	0.21	13.47	0.33	0	1.03	0.52	211.43	0.89	0
S	1	0.03	0.03	0.99	0	0.3	0.08	0.08	4.94	0.06	0	0	0	1.6	0	0.19
GS	2	0.08	0.04	1.48	0.01	0.21	0.06	0.03	1.87	0.05	0.1	0	0	1.01	0	0.36
R	46	1.27	0.03		0.11		0.71	0.02		0.56		0.11	0		0.1	
T	51	11.35			1		1.26			1		1.15			1	

Supplementary Table S2. Number of *park*^{25} pupae developed in the fecal transfer and fecal culture experiments.

Treatment	Total pupae	Number of vials	Pupae/vial	%Het	%Hom
Control feces transfer	1592	45	35.4	70.8	29.2
park^{25} feces transfer	1408	45	31.3	73.8	26.2
Axenic	9646	176	54.8	70.4	29.6
Control fecal bacteria culture	2913	63	46.2	67.8	32.2
park^{25} fecal bacteria culture	2622	59	44.4	65.8	34.2

Supplementary Table S3. Statistics of fecal and fly microbiota composition from control and *park*[25] mutants.

Effects of fly genotype (G), sample type (S, feces or fly), and the G * S interaction (GS) are shown along with residuals (R) and totals (T) as determined by PERMANOVA. PERMANOVA values are degrees of freedom (df), sum of squares (SS), mean squares (MS), F statistic (F), R^2 value (R^2), and P-value (P).

		Bray Curtis					Unweighted Unifrac					Weighted Unifrac				
	df	SS	MS	F	R^2	P	SS	MS	F	R^2	P	SS	MS	F	R^2	P
G	1	1.09	1.09	11.98	0.12	0	0.26	0.26	2.26	0.05	0.04	0.04	0.04	5.88	0.07	0
S	1	3.91	3.91	43.02	0.42	0	1.49	1.49	12.9	0.27	0	0.3	0.3	43.07	0.49	0
GS	1	1.33	1.33	14.63	0.14	0	0.14	0.14	1.21	0.02	0.23	0.05	0.05	7.64	0.09	0
R	32	2.91	0.09		0.31		3.7	0.12		0.66		0.23	0.01		0.36	
T	35	9.24			1		5.59			1		0.62			1	

Supplementary Figure S1. Homozygous *park*^{25} pupation rates are reduced on two consecutive developmental days.

The percentage of pupae developed out of the 60 embryos placed in each tube for each genotype in the two different fecal transfer conditions was calculated on days 5, 6, 7, and 8 post-embryo collection. The heterozygous (Het) *park*^{25} pupae were differentiated from the homozygous (Hom) *park*^{25} pupae by the presence of the Tubby marker on the TM6C balancer chromosome. Statistics were performed on the ArcSin transformed percentages. Two-way ANOVA results were significant for all variables: fly genotype & feces, day, and interaction (all $P < 0.0001$). Data are presented as mean and SEM. Post-hoc Tukey's analysis for each fly genotype comparing *park*^{25} feces vs control feces pupation rate results are shown as asterisks: * = $P < 0.05$, ** = $P < 0.01$, **** = $P < 0.0001$. Results are from 45 separate vials.

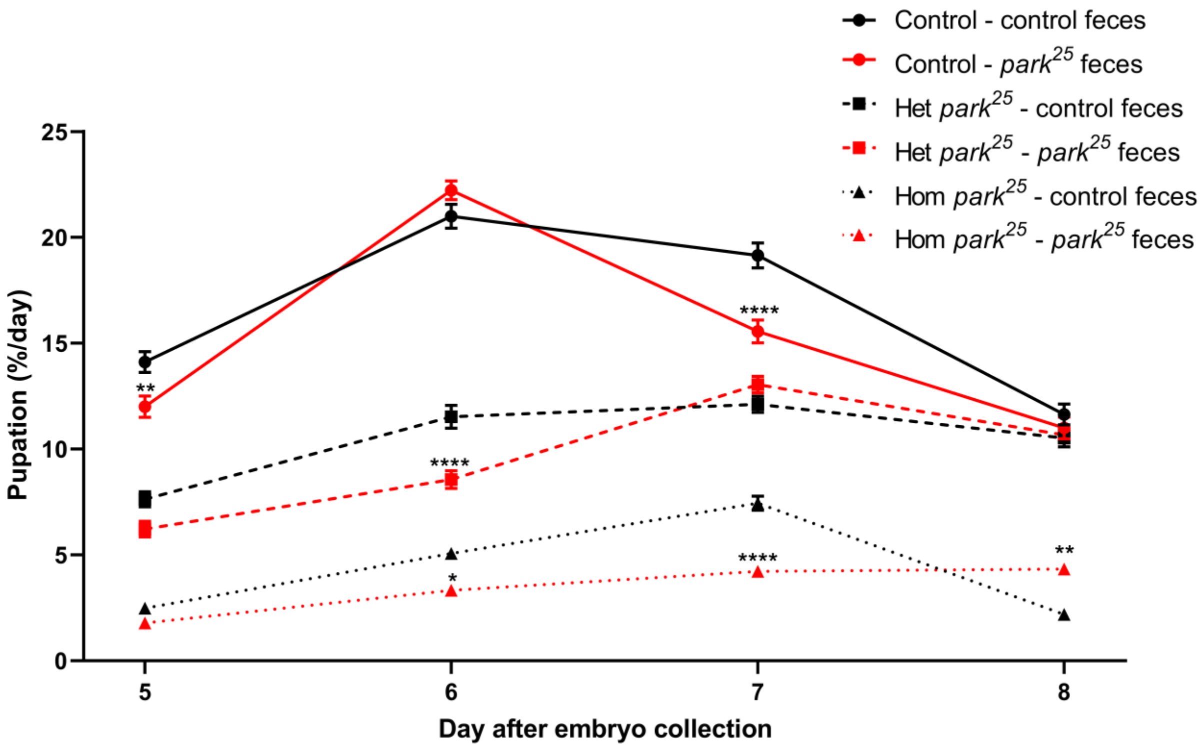

Supplementary Figure S2. Failure to eclose is increased in *park*^{25} flies only.

The average percentage of flies failing to eclose in each tube for each genotype in the two different fecal transfer conditions were calculated. The heterozygous (Het) *park*^{25} pupae were differentiated from the homozygous (Hom) *park*^{25} pupae by the presence of the Tubby marker on the TM6C balancer chromosome. Statistics were performed on the ArcSin transformed percentages. Data are presented as mean and SEM. Two-way ANOVA results were significant for all variables: fly genotype & feces, day, and interaction (all $P < 0.0001$). Post-hoc Sidak's tests comparing the effects of *park*^{25} vs control feces within genotypes on eclosion failure rates are shown as asterisks: ** = $P < 0.01$, **** = $P < 0.0001$. Results are from 45 separate vials.

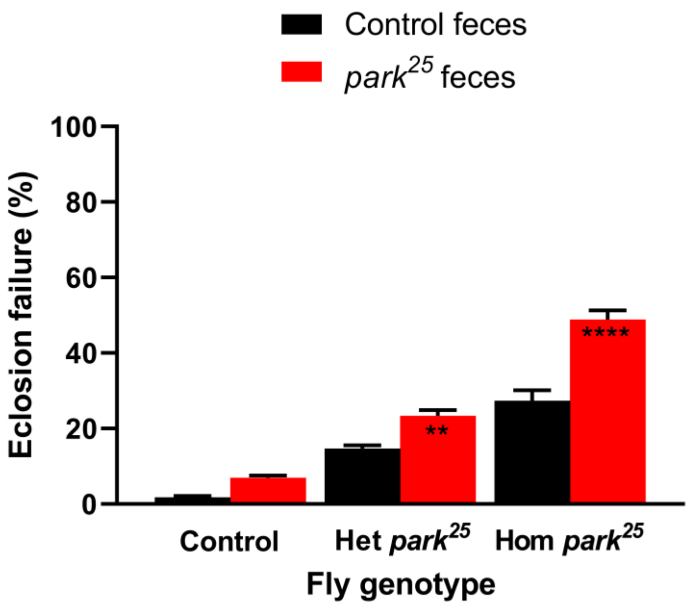

Supplementary Figure S3. Homozygous *park*^{25} flies have reduced eclosion three days in a row.

The percentage of **A)** control **B)** heterozygous *park*^{25} and **C)** homozygous *park*^{25} flies eclosing in each tube in the two different fecal transfer conditions was calculated. Statistics were performed on the ArcSin transformed percentages. Two-way ANOVA results were significant for all variables: fly genotype & feces, day, and interaction (all $P < 0.0001$). Data are presented as mean and SEM. Post-hoc Tukey's analysis of eclosion rates comparing the effects of *park*^{25} vs control feces results are shown as asterisks: ** = $P < 0.01$, *** = $P < 0.001$, **** = $P < 0.0001$. Results are from 45 separate vials.

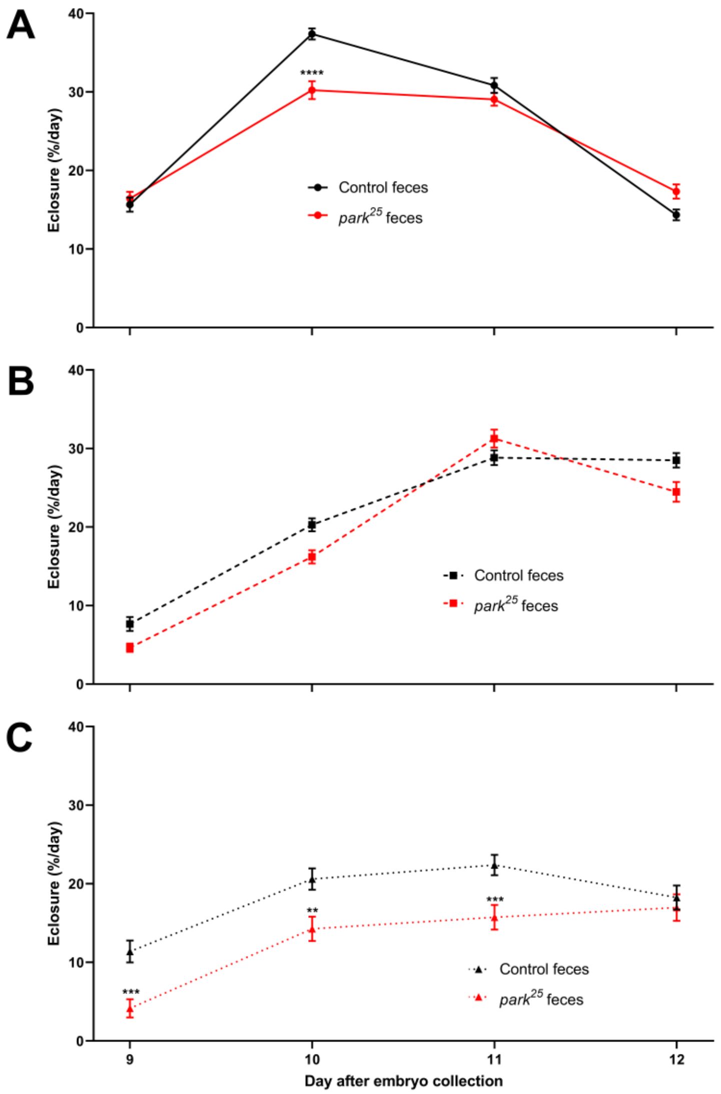

Supplementary Figure S4. The whole-body microbiota of control and *park*^{25} flies.

A reanalysis of data corresponding the samples presented in **Fig 3** before *Wolbachia* reads were removed. **A)** Taxon plot of control and *park*^{25} flies, separated by sex. Mutants of *park*^{25} were distinguished as homozygotes and heterozygotes based on the presence of the Tubby marker. Bars represent distinct ASVs. The legend shows the lowest taxonomic level that was assigned to each ASV. Principal coordinates plots, showing the first two coordinates calculated from a **B)** weighted Unifrac, **C)** unweighted Unifrac, or **D)** Bray Curtis distance matrix.

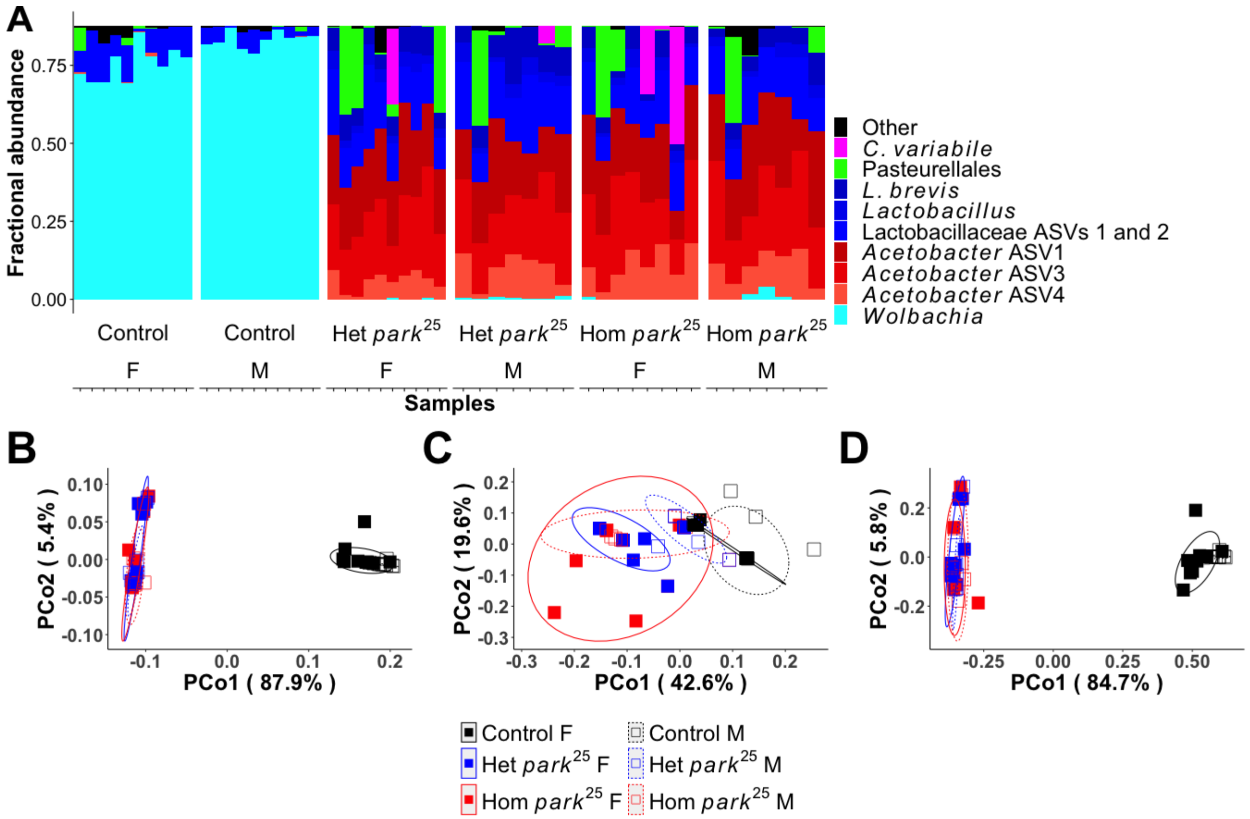

Supplementary Figure S5. Principal coordinates plots of Figure 3 data.

Principal coordinates plots of control and *park*^{25} flies from Figure 3, separated by sex. Mutants of *park*^{25} were distinguished as homozygotes and heterozygotes based on the presence of the Tubby marker in the TM6C balancer chromosome. Each plot shows the first two coordinates, calculated from **A)** a Bray Curtis or **B)** an unweighted Unifrac distance matrix.

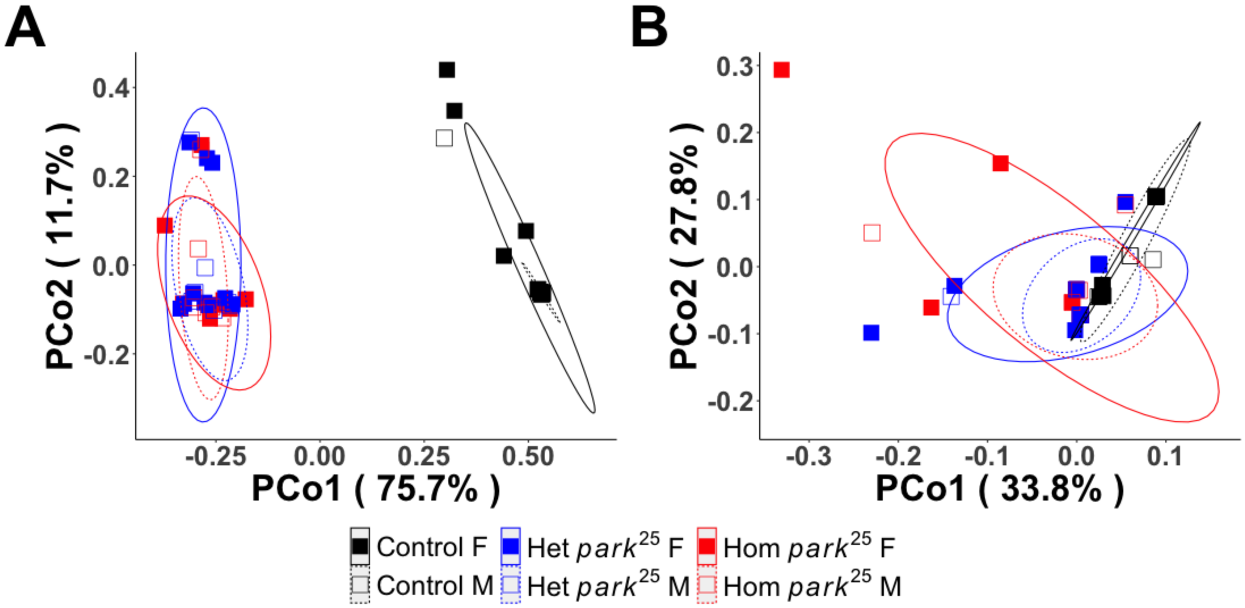

Supplementary Figure S6. Lactic acid bacteria and acetic acid bacteria are differentially abundant between different fly genotypes.

The relative abundance of bacterial ASVs that were differentially abundant between fly genotypes is shown (P < 0.05 by BH-corrected Analysis of Composition of Microbiomes [ANCOM] to test for differences in the abundances of specific individual or groups of ASVs[1], W statistics ≥ 10 in all cases, ANCOM does not perform post-hoc analysis, therefore no genotype comparison statistical results can be shown). Bar colors are the same as in Figure 3. The ASVs are all LAB (A-D) or AAB (E-H). Data are presented as mean and SEM. For all graphs, *n* = 15 for Hom *park*^{25}, 17 for Het *park*^{25}, 18 for control.

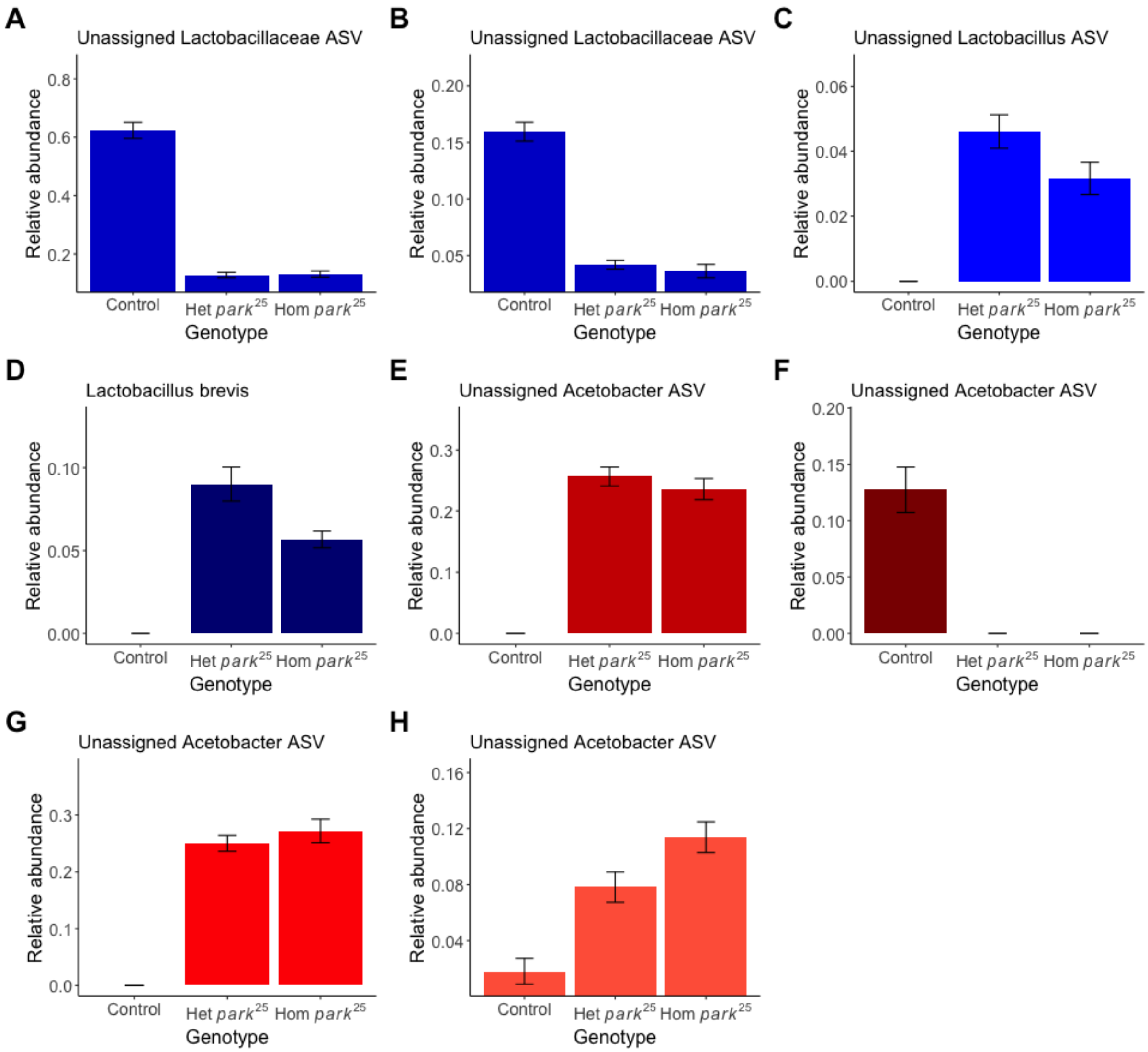

Supplementary Figure S7. Principal coordinates plots of Figure 5 data.

Principal coordinates plots of feces collected from control and *park*^{25} flies. Analysis is from the same data as shown in Figure 5. Each plot shows the first two coordinates, calculated from **A)** a Bray-Curtis or **B)** an unweighted Unifrac distance matrix.

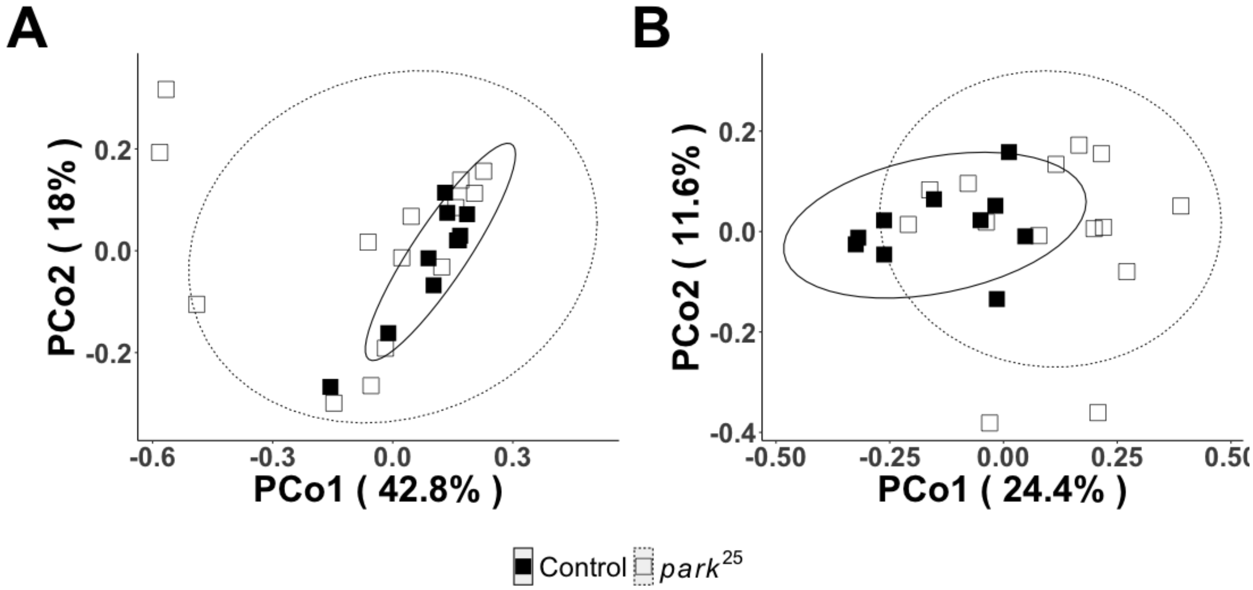

Supplementary Figure S8. The fecal and whole-body microbiota of control and *park*^{25} flies.

A reanalysis of data corresponding the samples presented in **Fig 5** plus additional close time-matched adult samples. **A)** Taxon plot of control and *park*^{25} flies (mixed sexes and genotypes [heterozygous and homozygous]). Bars represent distinct ASVs. The legend shows the lowest taxonomic level that was assigned to each ASV. Principal coordinates plots, showing the first two coordinates calculated from a **B)** weighted Unifrac, **C)** unweighted Unifrac, or **C)** Bray Curtis distance matrix.

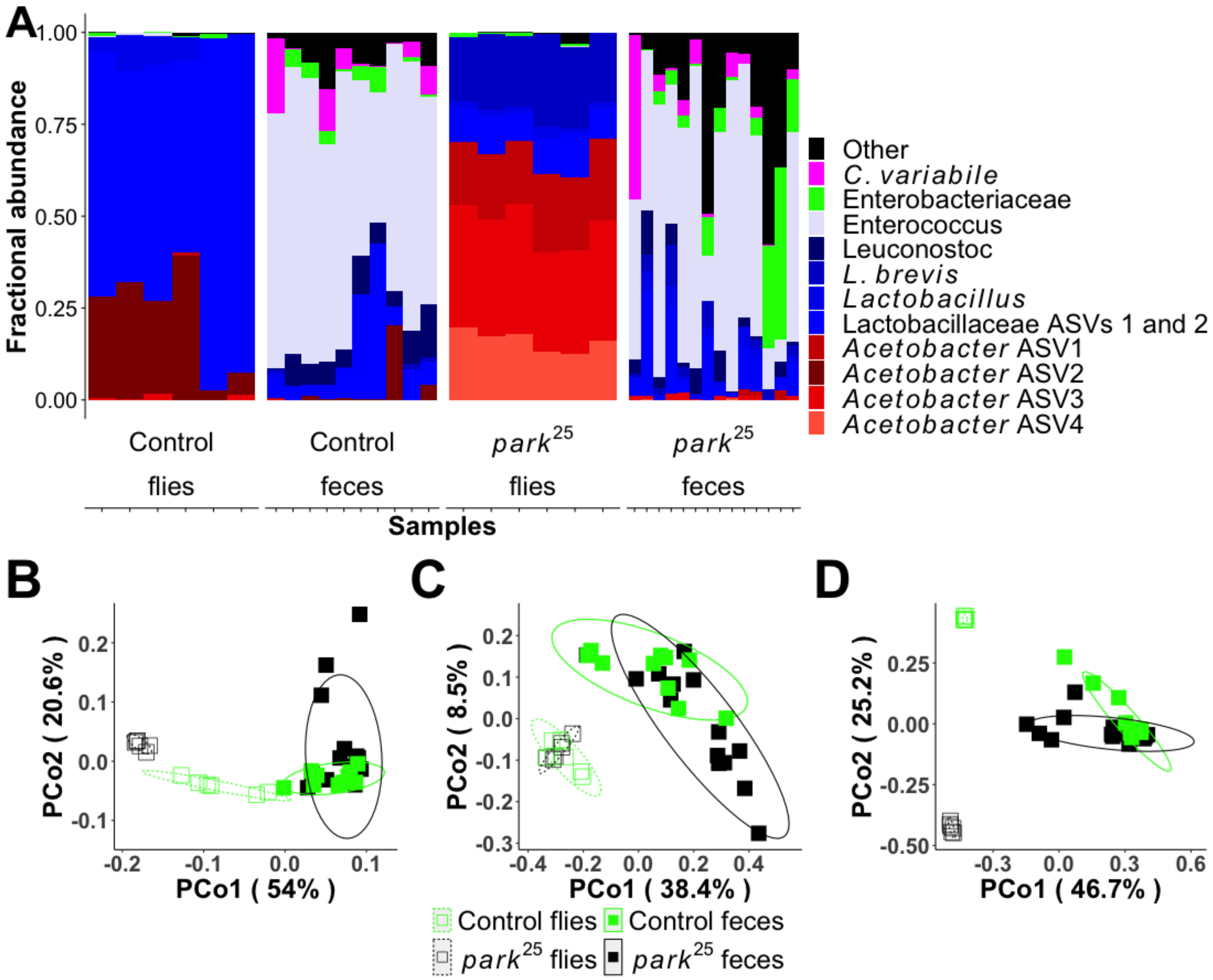

References

1 Mandal, S. *et al.* Analysis of composition of microbiomes: a novel method for studying microbial composition. *Microb Ecol Health Dis* **26**, 27663, doi:10.3402/mehd.v26.27663 (2015).
